# Novel mammographic image features differentiate between interval and screen-detected breast cancer: a case-case study

**DOI:** 10.1186/s13058-016-0761-x

**Published:** 2016-10-05

**Authors:** Fredrik Strand, Keith Humphreys, Abbas Cheddad, Sven Törnberg, Edward Azavedo, John Shepherd, Per Hall, Kamila Czene

**Affiliations:** 1 Department of Medical Epidemiology and Biostatistics, Karolinska Institutet, P.O. Box 281, Stockholm, SE-171 77 Sweden; 2 Department of Diagnostic Radiology, Karolinska University Hospital, Solna, Sweden; 3 Swedish eScience Research Centre (SeRC), Karolinska Institutet, Solna, Sweden; 4 Department of Cancer Screening, Stockholm-Gotland Regional Cancer Centre, Stockholm, Sweden; 5 Department of Molecular Medicine and Surgery, Karolinska Institutet, Solna, Sweden; 6 Department of Radiology and Biomedical Imaging, UCSF School of Medicine, University of California San Francisco, San Francisco, CA USA

**Keywords:** Breast cancer, Interval cancer, Cancer screening, Early detection, Mammography, Computer-assisted image processing

## Abstract

**Background:**

Interval breast cancers are often diagnosed at a more advanced stage than screen-detected cancers. Our aim was to identify features in screening mammograms of the normal breast that would differentiate between future interval cancers and screen-detected cancers, and to understand how each feature affects tumor detectability.

**Methods:**

From a population-based cohort of invasive breast cancer cases in Stockholm-Gotland, Sweden, diagnosed from 2001 to 2008, we analyzed the contralateral mammogram at the preceding negative screening of 394 interval cancer cases and 1009 screen-detected cancers. We examined 32 different image features in digitized film mammograms, based on three alternative dense area identification methods, by a set of logistic regression models adjusted for percent density with interval cancer versus screen-detected cancer as the outcome. Features were forward-selected into a multiple logistic regression model adjusted for mammographic percent density, age, BMI and use of hormone replacement therapy. The associations of the identified features were assessed also in a sample from an independent cohort.

**Results:**

Two image features, ‘skewness of the intensity gradient’ and ‘eccentricity’, were associated with the risk of interval compared with screen-detected cancer. For the first feature, the per-standard deviation odds ratios were 1.32 (95 % CI: 1.12 to 1.56) and 1.21 (95 % CI: 1.04 to 1.41) in the primary and validation cohort respectively. For the second feature, they were 1.20 (95 % CI: 1.04 to 1.39) and 1.17 (95%CI: 0.98 to 1.39) respectively. The first feature was associated with the tumor size at screen detection, while the second feature was associated with the tumor size at interval detection.

**Conclusions:**

We identified two novel mammographic features in screening mammograms of the normal breast that differentiated between future interval cancers and screen-detected cancers. We present a starting point for further research into features beyond percent density that might be relevant for interval cancer, and suggest ways to use this information to improve screening.

**Electronic supplementary material:**

The online version of this article (doi:10.1186/s13058-016-0761-x) contains supplementary material, which is available to authorized users.

## Background

Interval cancer (IC) is a cancer that is diagnosed in the interval between two mammographic screening visits. In this study, we have defined IC as breast cancer that is detected after a negative screen, or after a positive screen with a negative workup, but before the next regular visit or end of a normal screening interval, whichever came first. The length of a normal screening interval was between 18 and 24 months depending on age and county. IC has been shown to have a more aggressive phenotype than screen-detected cancer (SC) [1–7]. Several breast cancer screening strategies have been proposed which might decrease the IC incidence, such as increasing the screening frequency [8] or using supplemental imaging [9] in addition to mammography, e.g., magnetic resonance imaging (MRI) or ultrasound. However, adding examinations strain scarce medical resources, increase cost and impose a burden on the women in the screening program. There is a need to more accurately identify women at high risk of interval breast cancer and to understand which strategy would be most adequate for decreasing the risk of interval cancer by earlier screen detection.

Until now, the only feature of mammographic images of the normal breast that has been found to be associated with IC, compared with SC, is percent mammographic density (PD), i.e., the percentage of the pixels of the total breast area that has an intensity above a certain threshold. The association between high PD and interval cancer is believed to be related to an increased risk that dense benign tissue would mask a tumor [1, 7, 10]. If a woman has a high PD the tumor often needs to be larger before it can be detected on a screening mammogram [11, 12]. Previous studies of associations between interval cancer and image features other than PD have mainly focused on identifying pre-malignant changes [13, 14].

In the present study we examine a large set of image features, an approach that is often applied in machine learning. This feature-based approach has not been used previously in IC studies of the mammographic image of the normal breast phenotype. Our aim was to identify image features, in addition to PD, of preceding negative screening mammograms that might differentiate between interval cancers and screen-detected cancers. A secondary aim was to examine which feature might help us more accurately select women for whom supplemental imaging examinations would be most valuable in order to detect tumors earlier.

## Methods

### Study population

We analyzed breast cancer cases in the Libro-1 population-based cohort, which consists of women in the Stockholm-Gotland region diagnosed with breast cancer from 2001 to 2008. The Libro-1 cohort has been described in detail earlier [7, 15]. All women were identified through the Stockholm-Gotland Regional Breast Cancer quality register. Invitations were mailed out in 2009, together with informed consent documents and a link to an online questionnaire. The quality and organization of the Stockholm mammography screening program has been described in detail previously by Lind et al. [16]. For our study, only women diagnosed with incident unilateral invasive breast cancer, without any other previous cancer with the exception of non-melanoma skin cancer, without prior breast surgery, for which mode of detection was available, and who had at least one pre-diagnostic mammogram and corresponding PD measurement were included (*n* = 1403). Our primary study sample consisted of 394 IC cases and 1009 SC cases.

To try to validate our findings, we analyzed a second population-based cohort of breast cancer cases, called ‘CAHRES’, which has been described in detail elsewhere [17]. It contains incident breast cancer cases diagnosed from October 1, 1993, to March 15, 1995, and reported to any of the six Swedish Regional Cancer Registries. The validation sample included 1182 breast cancer cases (281 IC and 901 SC). The same inclusion and exclusion criteria were applied as for the primary cohort.

### Data collection

The data collection approach has been described in more detail previously [7]. Information about body mass index (BMI), hormone replacement therapy (HRT), and other sociodemographic, anthropometric, hormonal, and lifestyle factors were obtained through questionnaires collected during 2009. Use of HRT was classified as ‘yes’ if HRT pills had been used during the year of diagnosis, and as ‘no’ otherwise. Tumor characteristics were obtained from linkage with the Stockholm-Gotland Regional Breast Cancer quality register.

We collected mammograms by contacting local mammography units as well as the national Swedish medical image repository in Vilhelmina, Sweden. All mammograms were analog film mammograms that were digitized using an Array 2905HD Laser Film Digitizer, which covers a range of 0 to 4.7 optical densities. The density resolution was set at 12-bit dynamic range. The data collection was performed similarly for the validation cohort.

### Image analysis

All analysis was based on digitized film mammograms. Analysis was based on the last pre-diagnostic mammographic image per woman, using the mediolateral oblique view of the breast contralateral to the future tumor. To avoid image acquisition bias related to suspected or diagnosed cancer we did not consider mammograms that were acquired later than 30 days before diagnosis. PD values were calculated by an automated ImageJ-based method developed and validated by Li et al. in 2012 [18]. This method attempts to mimic the outcome of the gold standard area-based PD measurement method, Cumulus [19], and a high correlation (*r* = 0.884) between the two methods was demonstrated.

Our aim was to study features of the dense area in the mammograms, which were obtained by three processing steps: pre-processing of the image, dense area selection, and feature extraction. First, the images were pre-processed by automatically removing nametags and other artifacts, as well as reducing contrast intensity differences between mammograms, and finally by removing the image area corresponding to the pectoral muscle. Second, the dense area of the mammogram was automatically identified by a thresholding method. The dense area thus identified was not necessarily a single continuous area, but could consist of separate parts of the image. The primary thresholding approach was chosen to be Otsu’s method [20] for two reasons: it has been widely used and it was the only specified method used in the previous two studies of image features and interval cancer [14, 21]. In addition to Otsu’s method, we used two alternative thresholding methods: the percentile method and the entropy of histogram method [22]. Otsu’s method was based on minimizing the weighted sum of within-class variances of the foreground and background pixels to establish an optimum threshold. The percentile method was based on defining the dense area as all pixels with intensity above the median, i.e., the 50^th^ percentile. The entropy of the histogram method is based on maximizing the entropy of the histogram, which can be interpreted as maximum information transfer.

The third step in the image analysis involved the calculation of 32 different features based on the identified dense area. These were the same features as in a previous study from our group, which aimed to predict percent density from statistical features of digital mammograms (using machine learning approaches) [23]. That study showed that a density estimate based on feature extraction from processed digital mammograms was associated with breast cancer status, with a similar amount of evidence as that found for density measures calculated using another automated method (Volpara) on raw digital images. In the above-mentioned previous study, the features were selected to represent various feature categories. The statistical image features are based on the intensity value of each pixel, and can belong to either first-order or higher-order statistics. First-order statistics are based on the histogram of all pixels; examples include median, skewness and kurtosis. Higher-order statistics take the spatial relationship between pixels into account; examples include shape features based on fitting a shape to the silhouette of the segmented area and texture features based on measurements of the difference between neighboring pixels. The features were calculated using the computer software Matlab [24]. There were only two mammograms for which the quality did not allow feature calculations; one interval cancer and one screen-detected cancer.

### Statistical analysis

Before performing any statistical tests, we transformed the feature measures using a Box-Cox transformation after which they were standardized, in order to attain near standard normal distributions. We carried out 3 × 32 tests of association – three tests (the three thresholding methods described above) for each of the 32 extracted image features (Table 2). These (Wald) tests were based on fitting logistic regression models with IC versus SC status as the outcome and using continuous PD as an adjustment variable. We performed a global test of association testing the null hypothesis that none of the features were associated with IC versus SC status by examining the number of test results that were significant at the 5 % level (global test statistic). An empirical (global) level of significance was obtained by permuting IC versus SC status over a large number of simulations (10,000), and calculating the fraction of (global) test statistic values based on permuted data that were larger than the test statistic value obtained for the non-permuted data set. This global test is similar to Wilkinson’s test [25] but accounts for the correlation of the features.

To identify individual features, we used a forward selection procedure (the step function in R [26]) based on our logistic regression model with IC versus SC status as outcome and with continuous PD, age at diagnosis, BMI and HRT as covariates. The forward selection was based on features extracted from the dense area identified by our primary thresholding approach, Otsu’s method (see ‘Image analysis’). To test for potential confounding by different length of the time between mammography and diagnosis for IC and SC, we extended the final model by adding this as a covariate. The associations between the identified features and IC versus SC status were assessed in the validation cohort. Finally, we estimated univariate linear regression models with tumor size as the outcome and each feature as the predictor. Since tumor size was left-skewed we square-root-transformed the variable prior to analysis. The same analysis was performed within the SC and the IC subgroups separately. Additionally, regression models were fitted to examine the association between each of the identified features and each risk factor listed in Table 1. All statistical tests were two-sided. Statistical analyses were carried out in R [26] or Stata [27].

**Table 1:** Patient, mammographic and tumor characteristics (primary study sample)

	Study sample (*n = 1403*)			
	1C (*n = 394*) n (%) or mean	SC (*n = 1009*) n (%) or mean	*p* value	Missing data
PATIENT CHARACTERISTICS				
Age at diagnosis	60.8	60.2	0.055	0 %
Menopausal at diagnosis			0.150	6.4 %
Yes	297 (81 %)	794 (84 %)		
No	71 (19 %)	151 (16 %)		
BMI	24.8	25.8	<0.001	3.2 %
HRT use at diagnosis			<0.001	5.1 %
No	280 (75 %)	802 (84 %)		
Yes	93 (25 %)	156 (16 %)		
Oral contraceptive use			0.145	3.1 %
No	78 (21 %)	239 (24 %)		
Yes	300 (79 %)	742 (76 %)		
Parity				
Nulliparous	61 (15 %)	158 (16 %)	0.997	2.2 %
Number of births, parous women	2.13	2.14	0.816	0 %
Age at first birth, parous women	25.7	25.4	0.341	0 %
Family history of breast cancer (first degree)			0.029	7.8 %
No	284 (77 %)	762 (82 %)		
Yes	84 (23 %)	162 (18 %)		
MAMMOGRAPHIC CHARACTERISTICS				
Time (months) from measure to diagnosis	15.3 (median)	24.6 (median)	<0.001	
Mammographic percent density	23.5 (median)	17.6 (median)	<0.001	
TUMOR CHARACTERISTICS	n (%) or mean	n (%) or mean		
Tumor size, mm	19.5	15.6	<0.001	7.3 %
Lymph node metastasis			<0.001	0.4 %
No	356 (91 %)	960 (96 %)		
Yes	37 (9 %)	45 (4 %)		
Tumor grade, Elston			<0.001	42 %
1	35 (16 %)	147 (25 %)		
2	109 (50 %)	323 (54 %)		
3	75 (34 %)	127 (21 %)		
ER status			<0.001	27 %
Negative	57 (19 %)	72 (10 %)		
Positive	239 (81 %)	659 (90 %)		
PR status			0.002	28 %
Negative	109 (38 %)	201 (28 %)		
Positive	181 (62 %)	520 (72 %)		

*p* values for difference between the 1C and SC group were calculated by two-sided *t* test for continuous variables; and by chi square tests for categorical variables. See Additional file 1: Table S1 for patient and mammographic data for the validation sample

*1C* interval breast cancer, *SC* screen-detected breast cancer, *BMI* body mass index, HRT hormone replacement therapy, *ER* estrogen receptor, *PR* progesterone receptor

## Results

We included 1403 women, 394 IC cases and 1009 SC cases, in the primary study sample (Table 1). The women with IC, compared with SC, had a significantly lower BMI, higher PD, more often had used HRT during the year before diagnosis, and more often had a positive family history of breast cancer. Compared with SC tumors, the IC tumors were larger, had more often lymph node metastasis, were of a higher Elston grade, and were more often hormone receptor negative. Corresponding patient and mammographic characteristics for the validation sample can be found in Additional file 1: Table S1.

**Fig. 1:**
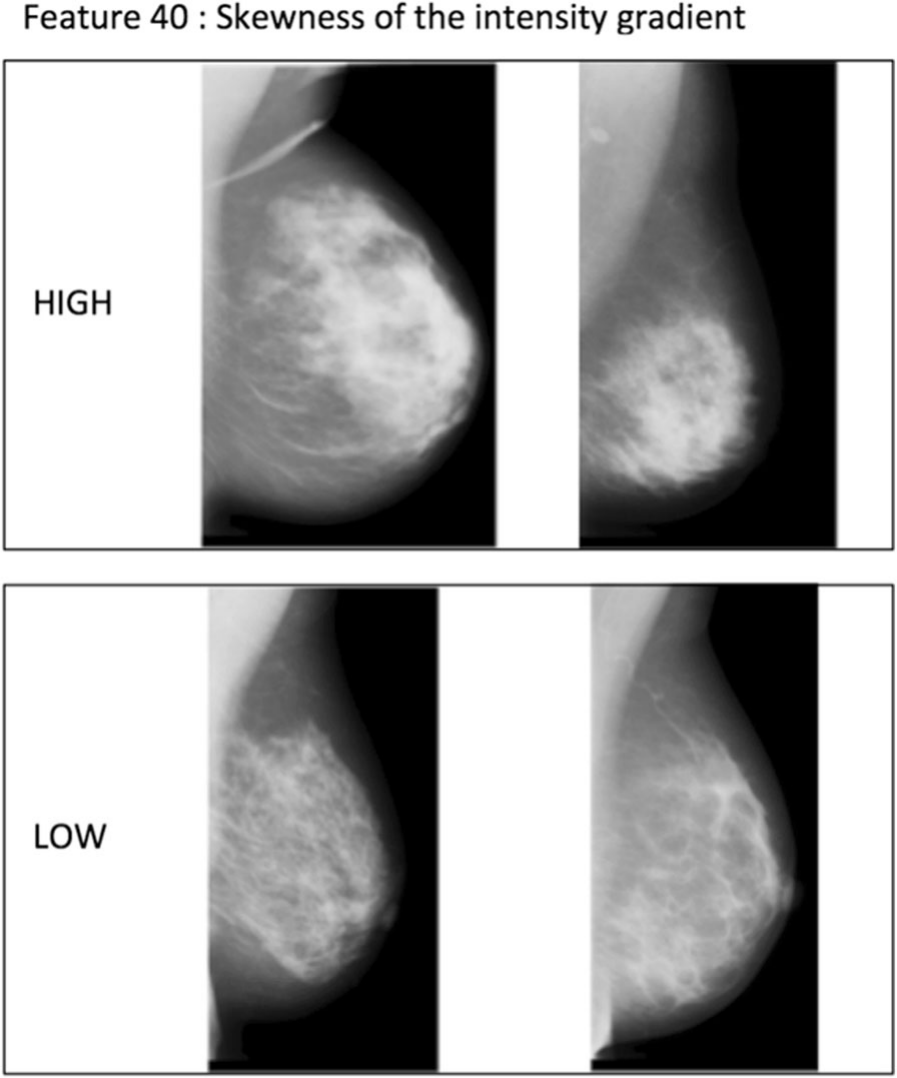
Feature F40 – ‘skewness of the intensity gradient’. Example of mammograms in our study which have a similar amount of dense area but differ in ‘skewness of the intensity gradient’. Our interpretation is that a high value corresponds to a more consolidated dense area, and a low value to a more scattered dense area

**Fig. 2:**
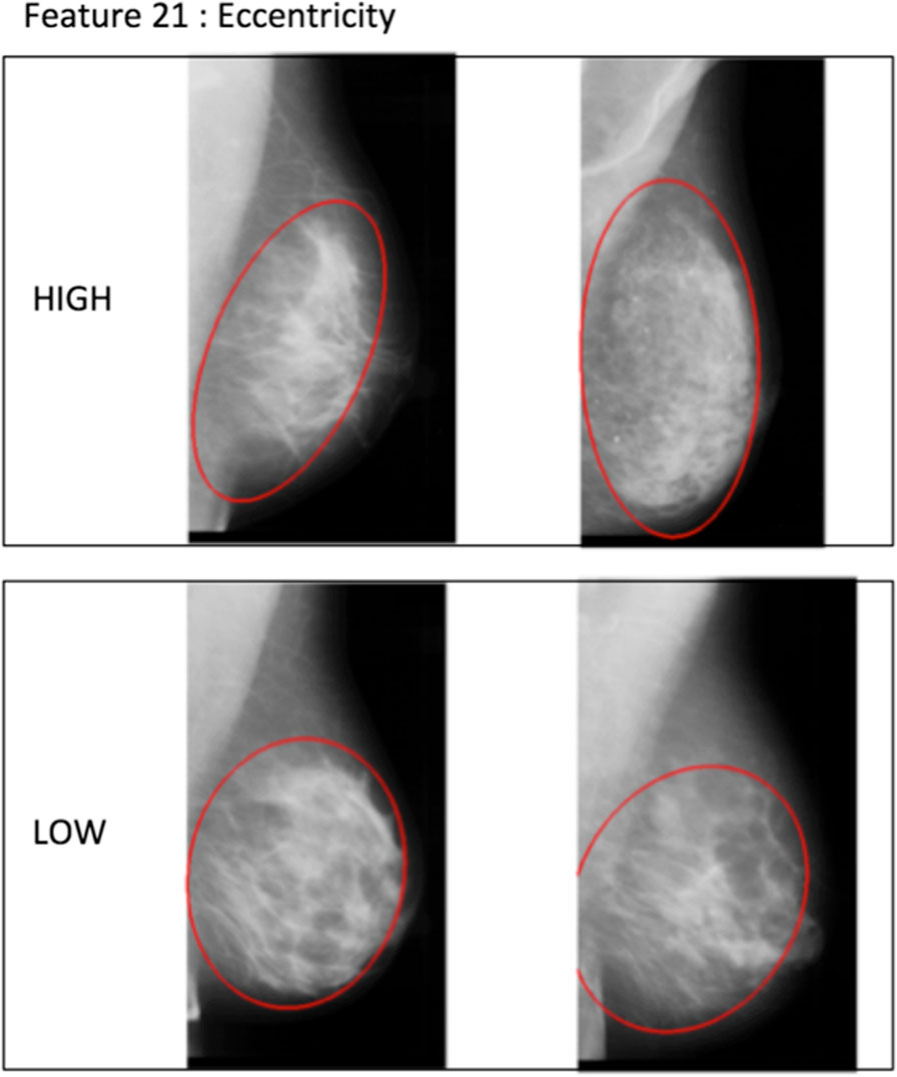
Feature F21 – ‘eccentricity’. Example of mammograms in our study which have a similar amount of total breast area and dense area but differ in ‘eccentricity’. Our interpretation is that a high value corresponds to a more elongated overall shape of the dense area, and a low value to a more circular shape

Individual *p* values for the associations between IC versus SC status and each combination of feature and dense area selection method are presented in Table 2, along with a short technical description of each feature. According to a global test of association we could refute the null hypothesis that none of the image features are associated with IC versus SC status with a *p* value of 0.007. Using a forward selection procedure, features ‘skewness of the intensity gradient’ and ‘eccentricity’ of the dense area were included in the final model. Eccentricity is calculated by fitting an ellipse to the dense area, and then taking the ratio of the distance between the foci of the ellipse and its major axis. Examples of mammograms with high and low ‘skewness of the intensity gradient’ are presented in Fig. 1, while examples of mammograms with high and low values of ‘eccentricity’ are presented in Fig. 2.

**Table 2:** *p* values for the association between each feature and IC versus SC, from logistic regression modelling, adjusted for mammographic percent destiny

	*p* value, *n* = 1401 (1C: 393, SC: 1008)			
	Dense area selection method			
Image feature	(1) Percentile	(2) Entropy	(3) Otsu's method	Technical feature description - all listed features are based on the identified dense area
F1	0.058	0.069	0.575	DC coefficient of two-dimensional discrete cosine transform
F2	0.636	**0.006**	0.233	The approximation coefficient of the multilevel discrete two-dimensional wavelet transform
F3	**0.002**	0.660	**0.006**	The max coefficient of the two-dimensional discrete Fourier transform of the local range filter
F4	0.193	0.697	**0.037**	Kurtosis of the intensity histogram
F5	0.388	0.334	0.053	Skewness of the intensity histogram
F6	0.074	**0.026**	**0.015**	Entropy of the intensity histogram
F7	0.282	**0.031**	0.969	The mean of entropy-filtered selected area
F8	0.467	**0.036**	0.793	Entropy of the complex imaginary part of the convolved selected area with log-Gabor filters
F9	0.624	**0.045**	0.969	Entropy of the complex real part of the convolved selected area with log-Gabor filters
F10	0.582	**0.025**	0.676	Entropy of the magnitude part of the convolved selected area with log-Gabor filters
F11	**0.020**	0.148	0.235	The max coefficient of the two-dimensional discrete Fourier transform
F12	0.995	**0.026**	0.234	Entropy of the normalized co-occurrence matrix
F14	0.600	0.751	0.326	The max coefficient of the two-dimensional discrete Fourier transform of the Hessian filter
F16	0.058	0.088	0.582	The max coefficient of the log of the magnitude part of the discrete cosine transform
F17	**0.020**	0.146	0.235	The max coefficient of the log of the magnitude part of the discrete Fourier transform
F18	0.111	0.078	0.443	The fourth central moment
F19	**0.012**	0.726	**0.019**	Number of separate blobs
F20	0.312	**0.015**	0.129	Solidity
F21	**0.001**	0.970	**0.007**	Eccentricity
F22	0.966	0.240	0.365	Euler number
F24	**<0.001**	0.931	**0.006**	Skewness of the normalized singular value decomposition/its standard deviation
F25	**0.040**	0.164	0.291	Singular value decomposition
F26	**0.062**	0.269	0.795	Number of holes within the selected area (low intensity surrounded by high intensity)
F27	0.063	**0.026**	**0.009**	Interquartile range of the intensity histogram
F33	0.225	0.317	0.322	Kurtosis of the projection along the Y axis
F34	0.065	0.136	0.201	Kurtosis of the projection along the X axis
F35	**0.002**	0.211	0.582	Perimeter of the selected area
F37	0.317	**0.040**	0.415	Mean intensity
F39	0.358	0.168	0.283	Median intensity
F40	0.145	**0.012**	**0.001**	Skewness of the intensity gradient
F41	0.172	**0.032**	0.946	Energy property of the co-occurrence matrix of the selected area - horizontal shift two pixels
F42	0.261	**0.031**	0.984	Energy property of the co-occurrence matrix of the selected area - diagonal shift eight pixels

*p* values in bold font signifies that they are below 0.05. Only features based on the dense area of the mammogram were included

*1C* interval breast cancer, *SC* screen-detected breast cancer

Table 3 presents the associations of the two image features and IC versus SC status in multivariate regression modelling. In the fully adjusted model, the per-standard deviation odds ratio (OR) for ‘skewness of the intensity gradient’ was 1.32 [95 % confidence interval (95 % CI): 1.12 to 1.56] in the primary cohort and 1.21 (95 % CI: 1.04 to 1.41) in the validation cohort, while the corresponding OR for ‘eccentricity’ was 1.20 (95 % CI: 1.04 to 1.39) and 1.17 (0.98 to 1.39). In the primary cohort, the median time periods between pre-diagnostic mammogram and diagnosis were 24.6 and 15.3 months for screen-detected and interval cancers respectively. Adjusting the final regression model for this time period only slightly changed the estimated odds ratios, and did not affect the significance of our results. Estimates of the association between each of the two identified features and the risk factors in Table 1 can be found in Additional file 2: Table S2.

**Table 3:** Associations between identified dense area features and IC versus SC based on multivariate logistic regression modelling

	Odds ratios (95 % Cl) for interval versus screen-detected cancer, estimated by logistic regression modelling			
	Primary cohort			Validation cohort
Covariate	Model 1 *n = 1403*	Model 2 *n = 1314*	Model 3 *n = 1312*	Model 3 *n = 1182*
Percent density	1.37 (1.22 to 1.53)	1.23 (1.08 to 1.41)	0.98 (0,81 to 1.17)	1.04 (0.86 to 1.24)
BMI	-	0.84 (0.73 to 0.98)	0.88 (0.76 to 1.02)	0.98 (0.84 to 1.15)
HRT use at diagnosis	-	1.57 (1.17 to 2.11)	1.53 (1.14 to 2.07)	1.32 (0.99 to 1.77)
Age at diagnosis	-	0.98 (0.87 to 1.12)	1.01 (0.89 to 1.15)	0.90 (0.77 to 1.05)
F40 skewness of the intensity gradient	-	-	1.32 (1.12 to 1.56)	1.21 (1.04 to 1.41)
F21 eccentricity	-	-	1.20 (1.04 to 1.39)	1.17 (0.98 to 1.39)

Feature values calculated based on the dense area of the mammogram as identified by Otsu's method, then Box-Cox transformed and standardized. Odds ratios are estimated as per-standard deviation change in the underlying covariate. Validation cohort is an older breast cancer cohort with similar covariate definitions as the primary cohort﻿﻿

*1C* interval breast cancer, *SC s*creen-detected breast cancer, *95 % CI* 95 % confidence interval, *BMI* body mass index, *HRT* hormone replacement therapy

As shown above, a high ‘skewness of the intensity gradient’ and a high ‘eccentricity’ were both associated with an increased risk of interval cancer compared with screen-detected cancer. From fitting linear regression models with tumor size as the outcome, separately for SCs and ICs, we found significant evidence that high ‘skewness of the intensity gradient’ of the dense area is associated with an increased tumor size for SCs, but not for ICs (Table 4). A high ‘eccentricity’ of the dense area was significantly associated with a decreased tumor size for ICs, but not for SCs (Table 4). A high ‘eccentricity’ of the dense area was significantly associated with a decreased tumor size for ICs, but without association with tumor size for SCs.

**Table 4:** Association between each identified feature and the tumor size, stratified by detection mode

	Beta coefficient (*p* value, 95 % confidence interval), estimated by linear regression modelling		
		Subgroup	
Image feature	All cancers (*n = 1299*)	Screen-detected (*n = 927*)	Interval cancer (*n = 372*)
F40 skewness of the intensity gradient	**0.16** (***p*** **< 0.001**, Cl: 0.09 to 0.23)	**0.20** (***p*** **< 0.001**, Cl: 0.12 to 0.29)	-0.05 (*p* = 0.465, Cl: -0.18 to 0.08)
F21 eccentricity	0.021 (*p* = 0.56S, Cl: -0.050 to 0.091)	0.055 (*p* = 0.188, Cl: -0.027 to 0.136)	**-0.18** (***p*** **= 0.014**, Cl: -0.32 to -0.04)

Tumor size was square-root transformed

Feature values calculated based on the dense area of the mammogram as identified by Otsu's method, then Box-Cox transformed and standardized beta coefficients represent the mean change in tumor size (measured in mm) per standard deviation change in transformed feature value Cl ***-*** 95 % confidence interval. *p* values in bold font signifies that they are below 0.05

## Discussion

We identified two novel mammographic features in screening mammograms of the normal breast that differentiated between future interval cancers and screen-detected cancers. Since we had no prior hypothesis about which features should be related to interval cancer, we examined a range of statistical and textural features used in a previous study [23] by our group. This set of features is not an exhaustive list of possible features to examine; many others have been used in mammographic studies previously [28, 29].

**Fig. 3:**
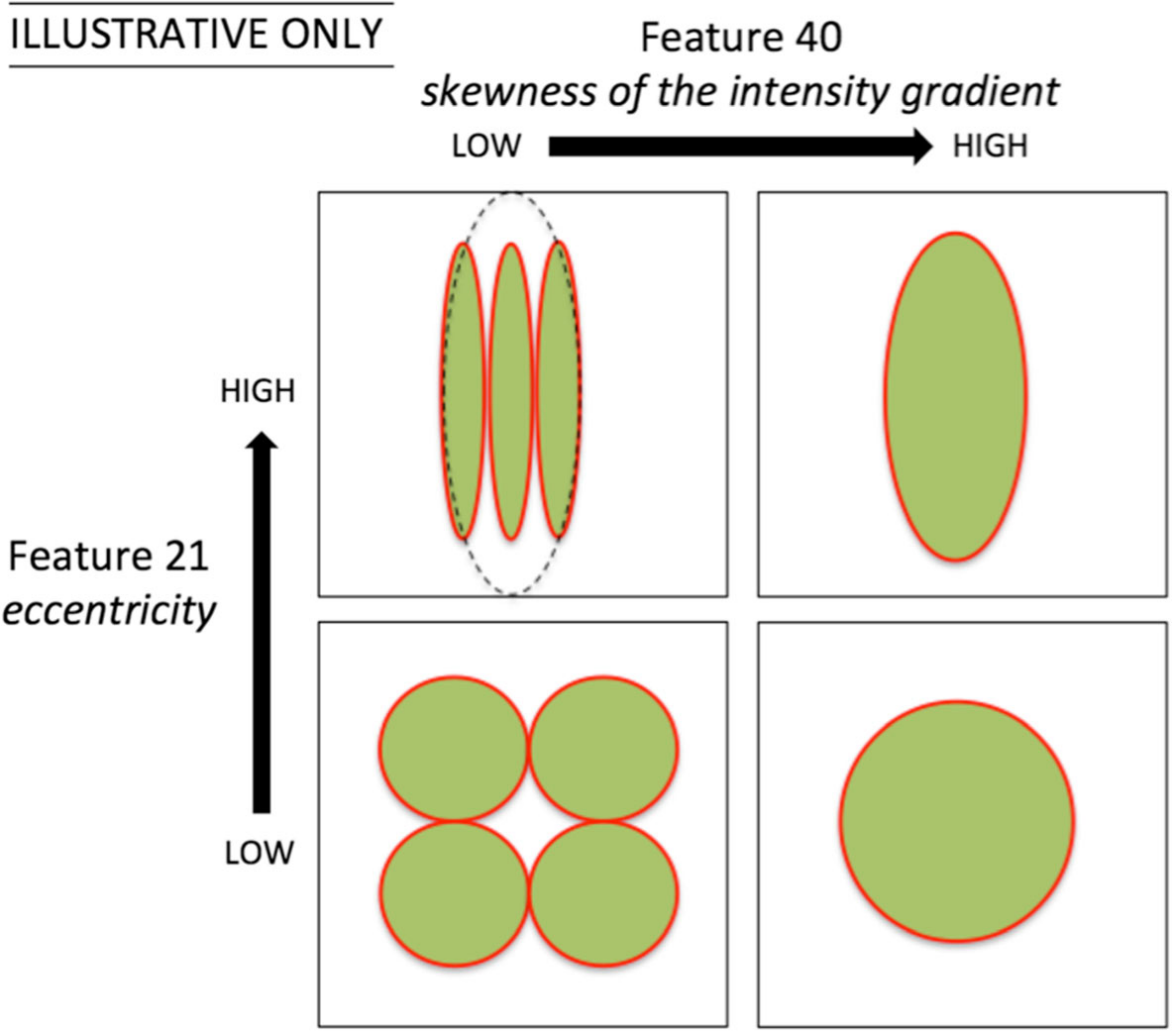
Illustration of combinations of the two identified features. Schematic images of four different dense areas with a high versus low value of ‘skewness of the intensity gradient’ and ‘eccentricity’

We identified two novel image features that were significantly different between interval and screen-detected breast cancers: first, the ‘skewness of the intensity gradient’, and second, the ‘eccentricity’ of the dense area in the mammogram. The two features are described in more technical detail in the Appendix. The *p* value for the association between certain features and IC versus SC status changed markedly depending on which thresholding method was used. This might be interpreted as either that certain features being more or less robust than others or that a specific thresholding method is better suited for calculating certain features. The two features that we identified were significant for two of three thresholding methods, and therefore considered to be quite robust. The first feature, after adjustment for percent density, was associated with a larger tumor size at screen detection, and thus related to a reduced mammographic detectability. We speculate that this feature is a reflection of the extent to which the dense area is interspersed by fatty streaks, reducing the size that a tumor must attain before being detectable at screening mammography. The second feature is related to how elongated the overall shape of the dense area is. This feature was associated with the size at interval cancer detection, most of which are detected by palpation. The mechanism why an elongated shape would promote early clinical detection is unclear. Speculatively, it might be related either to less firm normal breast tissue, or to tumors being located closer to the skin, both of which would enhance palpability. In Fig. 3, we illustrate how different combinations of high or low values of these two features may be manifested in an image.

Several breast cancer screening strategies to reduce the incidence of IC have been proposed, such as using a supplemental imaging [9], e.g., MRI or ultrasound, which would be most effective against masked tumors. If validated in a cohort including healthy women, the two mammographic features might increase our ability to individually tailor the breast cancer screening strategy. Supplemental imaging should potentially be directed toward women with a high percent density and a high value of our first feature ‘skewness of the intensity gradient’.

Previous studies of associations between interval breast cancer and image features include Banik et al. [14] who examined features potentially related to ‘architectural distortion’. The study was small, (56 interval cancer cases and 13 screen-detected cases) and did not take PD into consideration. Another study, by Tan et al. [21], examined the association between near-term breast cancer and the difference between image features of the left versus the right breast mammogram. Neither study included information about use of HRT, which is an established IC risk factor [7, 10]. Both studies were aimed at identifying pre-malignant changes, in principle masked tumors, while our study was aimed at features of the normal breast that would impact the risk of IC of any type: masked, fast-growing or facilitated clinical detection.

One strength of our study is that we were able to use an independent breast cancer cohort for the validation of the two features in a fully adjusted model. Another strength is that we not only examined image features but also tried alternative methods to select the dense area. We were also able to adjust for the potential risk factors PD and HRT, as well as age at mammography and BMI, with a relatively low degree of missingness. A potential weakness of our study is that BMI was measured several years after the analyzed mammographic images were acquired. This might have affected the (lack of) association between BMI and IC versus SC status in our final model. However, from previous literature there is no consistent evidence that such an association exists [30]. A limitation of our study is that we only examined digitized film mammograms. However, the same feature algorithms have been used previously for processed digital images, but for other purposes, and would thus need to be validated for digital mammograms in relation to IC versus SC status. The exact values of the features are the result of pre-processing, dense area selection, and feature calculation. This entire process was assessed in two independent cohorts yielding comparable odds ratios. In future studies aimed at validating our results, it would be important to perform all three steps according to our method.

## Conclusions

In conclusion, using a novel feature-based approach, we found two mammographic features of screening mammograms of the normal breast that differentiated between future interval cancers and screen-detected cancers independently of percent density. We present a starting point for further research into the utility of image features as a way to identify women at risk of interval cancer.

## Appendix – technical description of identified features

Feature F21 – ‘eccentricity’

The technical description of Feature 21 is eccentricity. Eccentricity was calculated by first fitting an ellipse according to the perimeter of the dense area based on the second moment method, and then calculating the ratio between the distance between the foci of the ellipse and its major axis. A single ellipse was fitted regardless of whether the dense area was one continuous shape or consisted of separate parts. Eccentricity is a continuous measure ranging from 0 to 1. A high eccentricity value means the ellipse is more elongated, less circular, and a low eccentricity value means the ellipse is rounder, more circular. All feature calculations were carried out in Matlab [24].

Feature F40 – ‘skewness of the intensity gradient’

The technical description of Feature 40 is the skewness of the intensity gradient. The intensity gradient is the change in intensity value between a certain pixel and its neighboring pixel. All pixels outside the selected dense area were defined as having zero intensity. The gradient in our analysis was based only on increases in the horizontal direction. When calculating the skewness, all non-dense area pixels are defined as having zero intensity. The largest gradient is thus achieved at the border between the dense area and the non-dense area. This gives the gradient value histogram a bimodal distribution, with the border pixels corresponding to large gradients and the interior pixels corresponding to smaller gradients. The intensity gradient is always skewed since there are more interior pixels than border pixels. The skewness is increased for a dense area having a smaller proportion of border pixels compared to interior pixels. All feature calculations were carried out in Matlab [24].

## Additional files

Additional file 1: Patient and mammographic characteristic (validation study sample). (DOC 440 kb)
Additional file 2: Association between risk factors listed in Table 1 and the two image features. (DOC 364 kb)

## Abbreviations

95 % CI: 95 % confidence interval
BMI: body mass index
HRT: hormone replacement therapy
IC: interval cancer
MRI: magnetic resonance imaging
OR: odds ratio
PD: percent (mammographic) density
SC: screen-detected cancer
