# Supplementary figures and images for: Novel mammographic image features differentiate between interval and screen-detected breast cancer: a case-case study
Source: Breast Cancer Res. 2016 Oct 5;18:100. doi: 10.1186/s13058-016-0761-x (PMC5053212; doi:10.1186/s13058-016-0761-x)

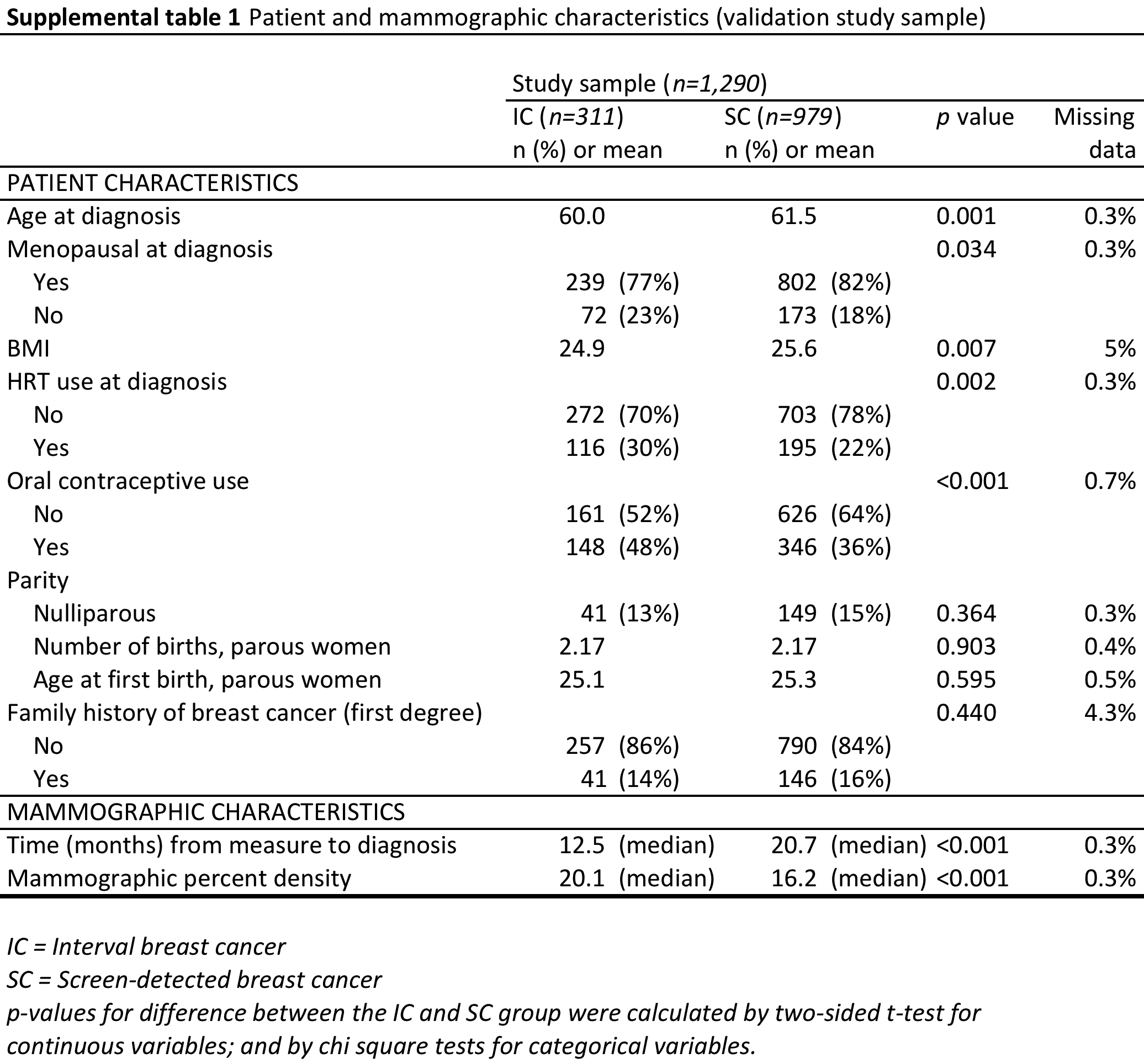

Supplement: Additional file 1: — Patient and mammographic characteristic (validation study sample). (DOC 440 kb) [file 13058_2016_761_MOESM1_ESM.doc]

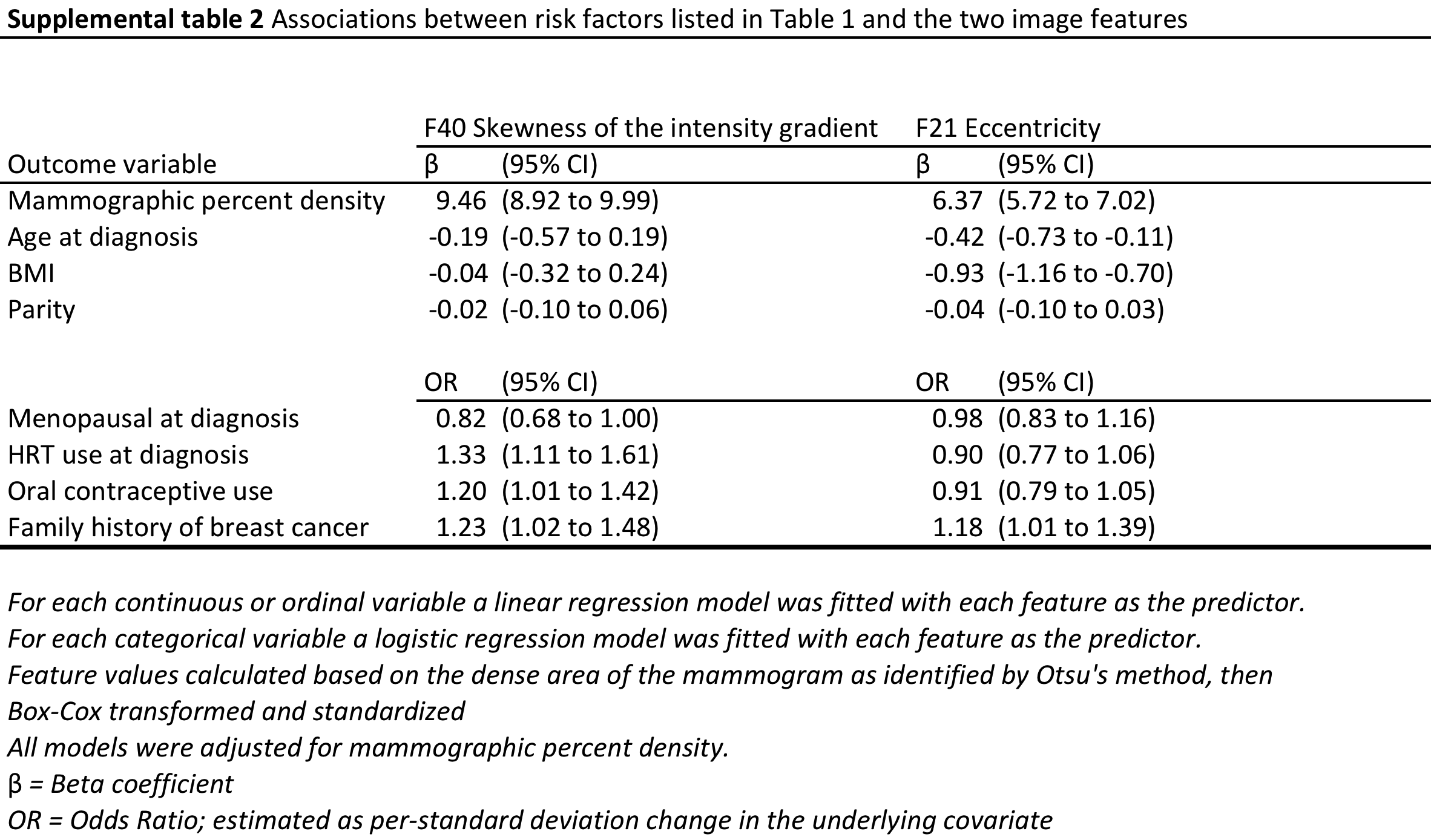

Supplement: Additional file 2: — Association between risk factors listed in Table 1 and the two image features. (DOC 364 kb) [file 13058_2016_761_MOESM2_ESM.doc]
